# Urine proteomics study reveals potential biomarkers for the differential diagnosis of cholangiocarcinoma and periductal fibrosis

**DOI:** 10.1371/journal.pone.0221024

**Published:** 2019-08-19

**Authors:** Kassaporn Duangkumpha, Thomas Stoll, Jutarop Phetcharaburanin, Puangrat Yongvanit, Raynoo Thanan, Anchalee Techasen, Nisana Namwat, Narong Khuntikeo, Nittaya Chamadol, Sittiruk Roytrakul, Jason Mulvenna, Ahmed Mohamed, Alok K. Shah, Michelle M. Hill, Watcharin Loilome

**Affiliations:** 1 Department of Biochemistry, Faculty of Medicine, Khon Kaen University, Khon Kaen, Thailand; 2 Cholangiocarcinoma Research Institute, Khon Kaen University, Khon Kaen, Thailand; 3 QIMR Berghofer Medical Research Institute, Brisbane, Australia; 4 Faculty of Associated Medical Sciences, Khon Kaen University, Khon Kaen, Thailand; 5 Department of Surgery, Faculty of Medicine, Khon Kaen University, Khon Kaen, Thailand; 6 Department of Radiology, Faculty of Medicine, Khon Kaen University, Khon Kaen, Thailand; 7 Proteomics Research Laboratory, Genome Institute, National Center for Genetic Engineering and Biotechnology, National Science and Technology Development Agency, Pathum Thani, Thailand; Texas A&M University, UNITED STATES

## Abstract

Cholangiocarcinoma (CCA) is a primary malignant tumor of the epithelial lining of biliary track associated with endemic *Opisthorchis viverrini* (Ov) infection in northeastern Thailand. Ov-associated periductal fibrosis (PDF) is the precancerous lesion for CCA, and can be detected by ultrasonography (US) to facilitate early detection. However, US cannot be used to distinguish PDF from cancer. Therefore, the objective of this study was to discover and qualify potential urine biomarkers for CCA detection in at-risk population. Biomarker discovery was conducted on pooled urine samples, 42 patients per group, with PDF or normal bile duct confirmed by ultrasound. After depletion of high abundance proteins, 338 urinary proteins were identified from the 3 samples (normal-US, PDF-US, CCA). Based on fold change and literature review, 70 candidate proteins were selected for qualification by multiple reaction monitoring mass spectrometry (MRM-MS) in 90 individual urine samples, 30 per group. An orthogonal signal correction projection to latent structures discriminant analysis (O-PLS-DA) multivariate model constructed from the 70 candidate biomarkers significantly discriminated CCA from normal and PDF groups (P = 0.003). As an independent validation, the expression of 3 candidate proteins was confirmed by immunohistochemistry in CCA tissues: Lysosome associated membrane glycoprotein 1 (LAMP1), lysosome associated membrane glycoprotein 2 (LAMP2) and cadherin-related family member 2 (CDHR2). Further evaluation of these candidate biomarkers in a larger cohort is needed to support their applicability in a clinical setting for screening and monitoring early CCA and for CCA surveillance.

## Introduction

Cholangiocarcinoma (CCA) is a primary malignant tumor of the epithelial lining of biliary track with high incidence in the northeastern Thailand where it is a major public health problem. Most CCA cases are clinically silent and difficult to detect at an early stage which leads to a poor prognosis and high mortality rates [1]. The major cause of CCA in the endemic area is liver fluke, *Opisthorchis viverrini* (Ov), infection. Ov infection can induce chronic inflammation, oxidative/nitrative stress, DNA damage, abnormal tissue remodeling, and alteration of gene expression which lead to periductal fibrosis (PDF) of the epithelial bile duct lining cells as a precursor to CCA [2–4]. PDF can be diagnosed by abdominal ultrasonography (US) and confirmed using CT/MRT and histology [5]. However, there is an urgent need for easily accessible biomarkers for differential diagnosis of CCA from PDF patients to enable early detection in high risk populations [6].

Arguably, analysis of bile for the discovery of CCA biomarkers is the ideal strategy as cancer cells are likely to release and/or secrete cancer-related proteins into bile [7]. However, bile is difficult to obtain from patients and requires an invasive technique for sample collection. In contrast, urine is an attractive source for biomarker testing because it can be collected easily and non-invasively, in large volumes [8]. Urinary proteins have been reported to provide potential biomarkers for urological diseases such as acute kidney injury[9], bladder cancer [10] and diabetic nephropathy [11]. Furthermore, as urinary proteins are composed largely of filtered plasma proteins, the urine proteome has suggested to provide potential biomarkers for non-renal diseases such as cardiovascular [12], autoimmune [13], pre-eclampsia [14] and infectious diseases [15], as well as non-urological cancers such as colon [16], ovarian [17], lung cancer [18] and CCA [19].

For CCA biomarkers, Metzger and co-workers developed a urine peptide marker model to differentiate CCA from primary sclerosing cholangitis (PSC), suggesting potential for non-invasive screening of CCA using urine. PSC is a risk factor for CCA in Western countries but not for Ov-associated CCA in Thailand. Thus, the aim of this study is to investigate potential biomarkers for CCA detection in urine samples of an at-risk population who were diagnosed with PDF using US in an endemic area in Thailand. We implemented a multi-phase study design, starting with shotgun proteomics for discovery of candidate biomarker proteins, followed by targeted proteomics via multiple reaction monitoring mass spectrometry (MRM-MS) for relative protein qualification, bioinformatics analysis for protein networks and immunohistochemistry for biomarkers validation in CCA human tissues.

## Materials and methods

### Study subjects and sample collection procedure

Participants supplying urine samples were selected from cohort studies conducted at the Cholangiocarcinoma Research Institute (CARI), in Khon Kaen, Thailand[20]. The ethics of experimentation using human specimens, based on the National Research Council of Thailand (HE531320 and HE571283) recommendations, were approved by the Human Ethics Committee of Khon Kaen University, Thailand. Written informed consent was obtained from all subjects in these studies. Inclusion criteria were: age 40 years or more, a family history of CCA, a history of Ov infection, negative to both hepatitis and cirrhosis, not pregnant or breast feeding, and residence in the endemic area of Ov infection. Abdominal ultrasonography (US) screening was used to classify participants in an endemic area for liver fluke infection in Khon Kaen province, northeast Thailand, into PDF-US or normal-US groups[5]. Subject in the PDF-US group are followed by US surveillance every 6 months [20]. CCA diagnosis were based on clinical data, imaging analysis and pathology. In this series, the anatomical classification of CCAs was classified as intrahepatic- (73.8%), perihilar- (11.9%), and extraductal- (14.3%) -type CCA. All mid-stream, random urine specimens from the first or second morning void were collected into a sterile screw-top plastic container and kept on ice. Centrifugation at 1,000 x g for 10 min within 2 hr was used to remove particulate matter. The supernatant was stored in aliquots of ~1mL in Eppendorf tubes at -80°C. Proteinuria was determined using the urine strip test (AUTION stick 10EA, Arkray, Japan). Tumor tissue microarrays (TMA) were obtained from 249 CCA patients who underwent liver resection at Srinagarind Hospital, Khon Kaen University, Thailand.

### Urine protein concentration and depletion

Urine samples were thawed on ice. They were then centrifuged at 1,000 × g for 30 min at 4°C and the urine supernatants concentrated using an Amicon Ultra-5 centrifugal filter with a 30 kDa molecular-weight cutoff (MWCO) (Millipore, VIC, Australia) at 3,200 × *g* to a volume of ~50 μL. The top 12 most abundant proteins (Thermo Fisher Scientific, MA, United States) were removed using an immunodepletion kit according to the manufacturer’s instructions. Concentrated urine (10 μL) was loaded onto the columns before being incubated with end-over-end mixing at room temperature for 1 h. The columns were then placed into a 2 mL collection tube and centrifuged at 1000 × g for 2 min. A Microcon-10kDa centrifugal filter unit with an ultracel-10 membrane at was centrifuged at 14,000 × g for 15 min (Ultracel-10 membrane, Merck Milipore, VIC, Australia) to concentrate the samples.

### Sample preparation for mass spectrometry analysis

The protein concentration in the immunodepleted urine was determined using a BCA kit (Thermo Fisher Scientific, MA, United States). A total of 30 μg of depleted urinary protein was denatured with 2% SDS in 100 mM triethylammonium bicarbonate (TEAB) at pH 8.5 and 95°C for 5 min. This was then reduced using 10 mM of Tris (2-carboxyethyl) phosphine (TCEP) at 60°C for 30 min before being alkylated with 40 mM 2- chloroacetamide (CAA) at 37°C in the dark for 30 min. We used the methanol co-precipitation method for peptide tryptic digestion as previously described [21]. Acidification was then accomplished by adding 1% formic acid (FA) after which the resultant product was cleaned and enriched with Strata-x polymeric reversed phase cartridges (Phenomenex pn 8B-S100-AAK, NSW, Australia) according to the manufacturer’s instructions. The peptide mixture was then dried in a vacuum centrifuge and later re-suspended with 1% FA before being stored at -80°C prior to analysis.

### Liquid chromatography-tandem mass spectrometry (LC-MS/MS) and database searching

#### Shotgun proteomics for the discovery cohort

The analysis of the tryptic peptides was carried out using a nano ACQUITY UPLC system (Waters, Milford, US) coupled to a Triple TOF 5600 mass spectrometer (AB SCIEX) that was equipped with a nano-electrospray ion source. The peptides were loaded on to a trap column M-Class 5 μm Symmetry C18 180 μm x 20 mm (Waters) and separated using an M-Class 1.7 μm BEH130 C18 75 μm x 200 mm LC column (Waters) with a flow rate of 300 nL/min and a column temperature of 35°C. There were 2 mobile phases: A with 0.1% FA in milliQ water and B with 0.1% FA in acetonitrile. The following gradient of mobile phase B was used: 1% at 0 min; 5% at 2 min; 25% at 75 min; 40% at 80 min; 95% at 85 min; 95% at 90 min; 2% at 92 min; 2% at 120 min. Information-dependent acquisition (IDA) mode was used in the Triple TOF5600 with a survey scan mass range of m/z 350–1,250 and a charge of 2–4 above the threshold of 70 cps being selected for fragmentation and then exclusion for 15 sec. The TripleTOF 5600plus mass spectrometer had an approximate resolution of 35,000 when operated in positive ion and high-resolution mode. The precursor selection mass window in the quadrupole was set to unit resolution (m/z 0.7 window). The following parameter settings were used for the ion source: gas 1 = 21, gas 2 = 0, curtain gas = 25, ion spray voltage = 2300 V. The ion optics parameters were: declustering potential = 100 V, collision energy (survey scan) = 10 V. Rolling collision energy voltage was used for CID (collision-induced dissociation) fragmentation with a collision energy spread of 3. For the collision gas nitrogen was used. Each cycle consisted of a TOF-MS survey scan (mass range: 350–1250 Da, dwell time: 250 ms). This was followed by sequential fragmentation (mass range: 50–2000 Da, accumulation time: 120 ms) of the 25 most intense precursors selected according to IDA criteria.

Quantitative label-free analysis of the LC-MS/MS data used MaxQuant software (version 1.6.1.0)[22, 23]. Raw data were scanned from the human Uniprot fasta database (20,202 entries, downloaded on July, 2017), while a common contaminants database (247 entries) was determined using the Andromeda search engine [24]. Fixed modification was Cysteine carbamidomethylation and variable modifications were N-terminal acetylation, deamidation at NQ, and methionine oxidation. Enzyme specificity was trypsin with a maximum of two missed cleavages and a minimum peptide length of seven amino acids. Peptide identifications were matched across all samples within a time window of 1 min of the aligned retention times. Peptide identification was performed with an allowed initial precursor mass deviation of up to 7 ppm and an allowed fragment mass deviation of 20 ppm. A rate of 1% for false discovery was applied at the peptide and protein levels. Identifications were matched across all samples with a time window of 1 min of the aligned retention times. A ‘match between runs’ library was constructed in MaxQuant using single shot MS runs. Protein identification required at least 1 ‘razor peptide’ in MaxQuant. The data were then filtered for common contaminants and the peptides identified by side modification only, those with unique peptides less than two and a score of protein identification less than five being excluded from further analysis.

#### Targeted proteomics for the qualification cohort using multiple reaction monitoring MS (MRM-MS)

A triple quadrupole mass spectrometer, LCMS-8050 (Shimadzu), coupled with a Shimadzu Nexera X2 ultra-high-performance liquid chromatograph (UHPLC) was used for MRM_MS analyses. Details of the method development are provided in S1 Appendix. A reversed-phase column AdvanceBio Peptide Mapping (150 × 2.1 mm i.d., 2.7 μm, part number 653750–902, Agilent Technologies) connected to a 5 mm guard column of the same material was used for peptide separation. Mobile phase A consisted of 0.1% FA and mobile phase B consisted of 0.1% FA in acetonitrile. Operating conditions were 60°C and a flow rate of 0.4 mL/min. The following gradient of mobile phase B was used: 3% at 0 min; 30% at 20 min; 40% at 24 min; 95% at 24.5 min; 95% at 28.5 min; 3% at 29 min; 3% at 34 min. Sample analysis was randomized, with the injection volume being set at 35 μL. The LC-MS 8050 triple quadrupole mass spectrometer was operated in positive ion mode using Labsolution software for control (version B.06.00 build 6.0.6025.4 SP4) with the Electrospray voltage set at 4.0 kV (positive ion), nebulizing gas flow 3 L/min, heating gas flow 10 L/min, drying gas flow 10 L/min, interface temperature 300°C, desolvation line temperature 250°C, and a heat block temperature of 400°C, unit resolution (0.7 Da full width at half maximum in the first quadrupole (Q1) and the third quadrupole (Q3)).

Skyline software (version 3.7.1.11208) (http://skyline.maccosslab.org/) was used to analyse the data [25]. All peaks were manually checked for correct integration and peptide intensity was defined as the peak area for each peptide based on the sum of all transitions. The data were then exported to R software (3.4.3 version) for analysis. A log2 transformation was performed to obtain a near-normal distribution. The peptide peak intensity was normalized using the median of normalized area under the peak of summed iRT peptides for each sample. Normalized peptides with correlations of >0.8 for each particular protein (Pearson’s product-moment correlation) were averaged to derive the normalized protein level. The missing (N/A) values were replaced with minimum normalized protein intensity detected over the entire experiment.

### Bioinformatics and statistical analysis

Pearson’s chi-square test using SPSS 19.0 (IBM, USA) was used to compare the clinical variables. In order to determine potential biomarkers multivariate analysis, principal component analysis (PCA) and orthogonal signal correction projection to latent structures discriminant analysis (O-PLS-DA) were conducted in SIMCA 15.0 (Umetrics, Sweden). Data normalization and statistical analyses were performed in Microsoft Excel and R software. Kruskal-Wallis tests (non-parametric distributions) were used to determine the differences of candidate proteins between groups. A value of *P*<0.05 indicated statistical significance.

### Immunohistochemical staining (IHC) for validation of candidate proteins

An independent set of archived formalin-fixed, paraffin-embedded sections of human CCA tissues were used to prepare tissue microarrays (TMAs) for IHC staining in the validation study. TMA sections were de-paraffinized with xylene and rehydrated using a stepwise decreasing concentration of ethanol for used immunohistochemical investigations. The antigen retrieval occurred by heating the slides in a microwave with 10 mM citrate buffer pH 6.0 with 0.05% Tween 20 at high power for 10 min. Endogenous H_2_O_2_ activity and non-specific binding were blocked by incubating the slides for 1 hr in 3% (v/v) hydrogen peroxide (H_2_O_2_) in PBS and for 1 hr with 10%(w/v) skim milk in PBS, respectively. Sections were then incubated with the primary antibody at 4°C overnight before being washed with 0.1% (v/v) Tween20 in PBS. The antibodies used were: LAMP1 (Cat. ab24170), LAMP2 (Cat. ab18528) purchased from Abcam (Cambridge, MA) and PCLKC (Cat. orb158119) purchased from Biorbyt (San Fransisco, CA). The sections were incubated with peroxidase-conjugated Envision^TM^ secondary antibody (Dako, USA), with the color being developed using a 3,3’diaminobenzidine tetrahydrochloride (DAB) substrate kit (Vector Laboratories, Inc., Burlingame, CA). Mayer’s haematoxylin was used for counter staining. The sections were rehydrated using a stepwise increasing concentration of ethanol before being mounted with Permount. The stained sections were scored under a microscope with a semi-quantitative scale based on percentages of positive cells being used to determine the staining frequency of the proteins: 0% = negative, 1–25% = 1, 26–50% = 2, and >50% = 3. The positive staining intensity was graded as weak = 1, moderate = 2, or strong = 3. The immunohistochemical (IHC) scores were calculated by multiplying the frequency score with the intensity score[26].

### Protein-protein interaction analysis

Protein interaction network analysis was carried out using STRING software (http://string-db.org) based on the STRING database and Gene Oncology (GO). Verified candidate proteins that were found to be correlated with CCA urine samples were subjected to a further protein-protein network analysis.

## Results

### The urine biomarker discovery workflow and baseline characteristics of subjects both discovery and qualification cohorts

**Fig 1:**
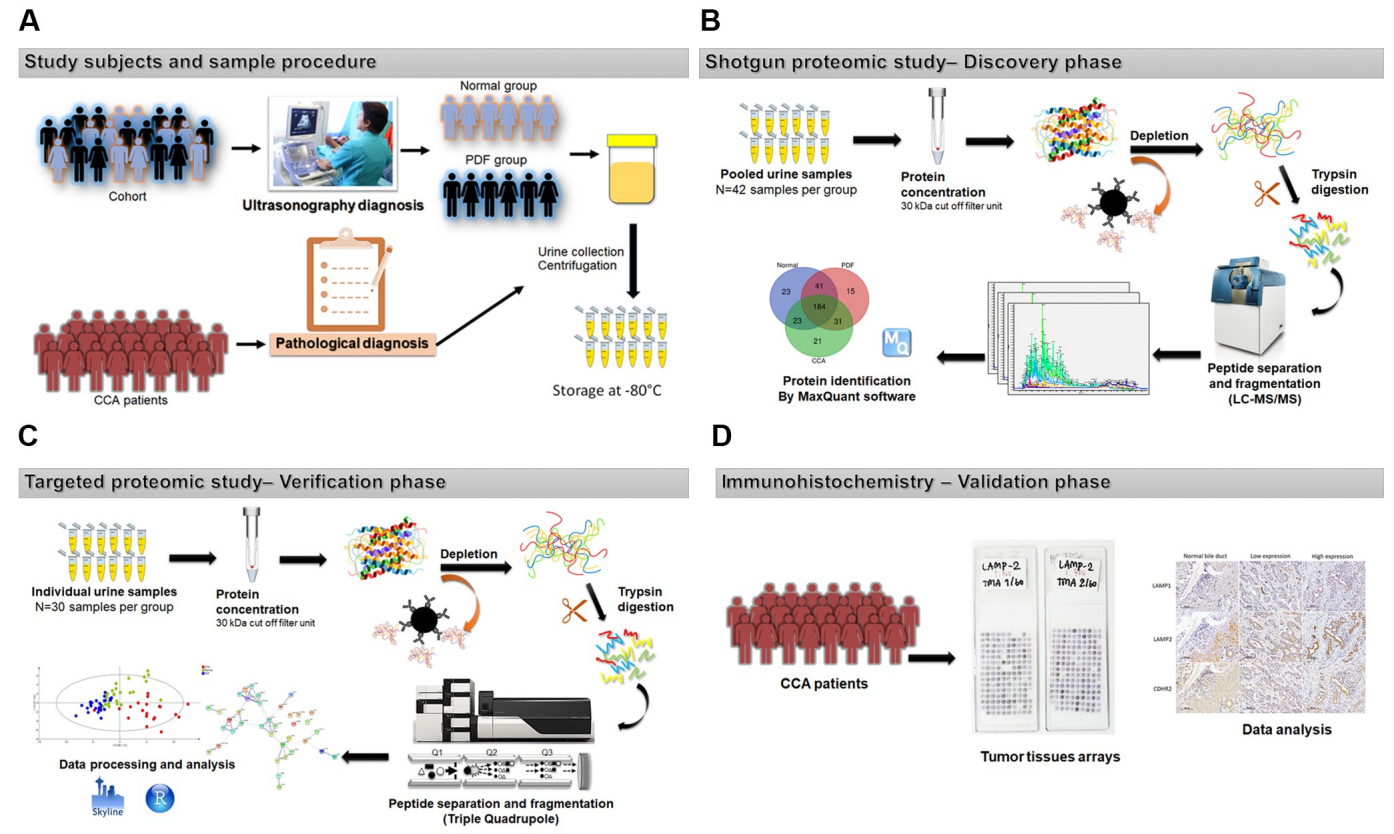
The workflow for urinary protein biomarker discovery. (A) urine samples were collected from participants and stored at -8000b0c until analysis. (B) urine samples selected for study were pooled, concentrated and depleted for highly-abundant protein removal using an immunodepletion kit, followed by in-solution tryptic digestion and tandem mass spectrometry. Protein identification and database searching were conducted using MaxQuant software (version 1.6.1.0). (C) Biomarker qualification for selected candidates used a custom MRM-MS assay. Data processing was performed using the Skyline and R programs. The statistical analysis software “SIMCA” and “SPSS" were used to perform the univariate/multivariate statistical analysis. (D) Immunohistochemical staining techniques were used to validate potential biomarkers on TMA.

Experimental design for the current urine biomarker discovery and qualification study is shown in Fig 1. From study cohorts at CARI, we selected 126 urine samples (42 normal-US, 42 PDF-US, 42 CCA) and age-sex matched urine samples from 90 individuals (30 normal-US, 30 PDF-US, 30 CCA) for the discovery and qualification phases, respectively (Table 1). Normal and PDF cases were confirmed using ultrasonography diagnosis by radiologists, while CCA was confirmed by pathology diagnosis. The clinical data were recorded for both the discovery and qualification phases, comprising the age, sex, proteinuria, smoking status, alcohol consumption and the pathological feature of CCA patients. The latter included tumor type and metastasis status as shown in Table 1. From the descriptive clinical data on the samples in the discovery and qualification cohorts, smoking status and alcohol consumption were more common in the CCA population when compared with the normal and PDF group. This is in agreement with a recent report found that smoking and alcohol consumption can increase the risk of hepatocellular carcinoma (HCC) and CCA development [27]. Proteinuria was significantly associated with normal and PDF groups for both cohorts (*P*<0.05, Table 1). The reason for this is unclear but there is a possible impact on proteomics data interpretation. Known CCA risk factors, smoking and alcohol consumptions was significantly associated with CCA group, as expected. in both the discovery and qualification cohorts (*P* = 0.003, Table 1).

**Table 1:** The clinical and demographic information on all subjects for the discovery and qualification groups.

Discovery phase				Qualification phase			
	Normal US	PDF US	CCA	*P*	Normal US	PDF US	CCA	*P*
	Sample size(n)	42	42	42		30	30	30	
**Sex**				1.000				1.000
Male	31 (74%)	31 (74%)	31 (74%)		19 (63%)	19 (63%)	19 (63%)	
Female	11 (26%)	11 (26%)	11 (26%)		11 (37%)	11 (37%)	11 (37%)	
**Age, year**								
Median±SD	63±5	62±6	65±6		63±5	62±4	64±7	
(Age range)	(55–73)	(55–77)	(43–76)		(55–73)	(55–69)	(43–76)	
**Proteinuria**				**0.046**				**0.036**
Positive	8 (19%)	7 (17%)	1 (2%)		5 (17%)	6 (20%)		
Negative	34 (81%)	35 (83%)	41 (98%)		24 (80%)	23 (77%)	0	
Unknown^‡^					1 (3%)	1 (3%)	30 (100%)	
**Smoking status**				**0.003**				**0.029**
Positive	14 (33%)	12 (29%)	27 (64%)		9 (30%)	7 (23%)	17 (57%)	
Negative	23 (55%)	26 (62%)	13 (31%)		17 (57%)	19 (63%)	11 (37%)	
Unknown^‡^	5 (12%)	4 (9%)	2 (5%)		4 (13%)	4 (14%)	2 (6%)	
**Alcohol consumption**				**<0.001**				**0.001**
Positive	13 (31%)	11 (26%)	33 (79%)		9 (30%)	7 (23%)	21 (70%)	
Negative	24 (57%)	27 (64%)	7 (17%)		17 (57%)	19 (63%)	7 (23%)	
Unknown^‡^	5 (12%)	4 (10%)	2 (4%)		4 (13%)	4 (14%)	2 (7%)	
**Histology type**								
Non-papillary			26 (70%)				18 (60%)	
Papillary			16 (30%)				12 (40%)	
**Metastatic status**								
No			22 (52%)				16 (53%)	
Yes			20 (48%)				14 (47%)	

NOTE

^‡^indicating there are missing data.

“***P***” indicating P-value were calculated using Pearson’s chi-square test.

### Urinary biomarker discovery using shotgun proteomics

**Fig 2:**
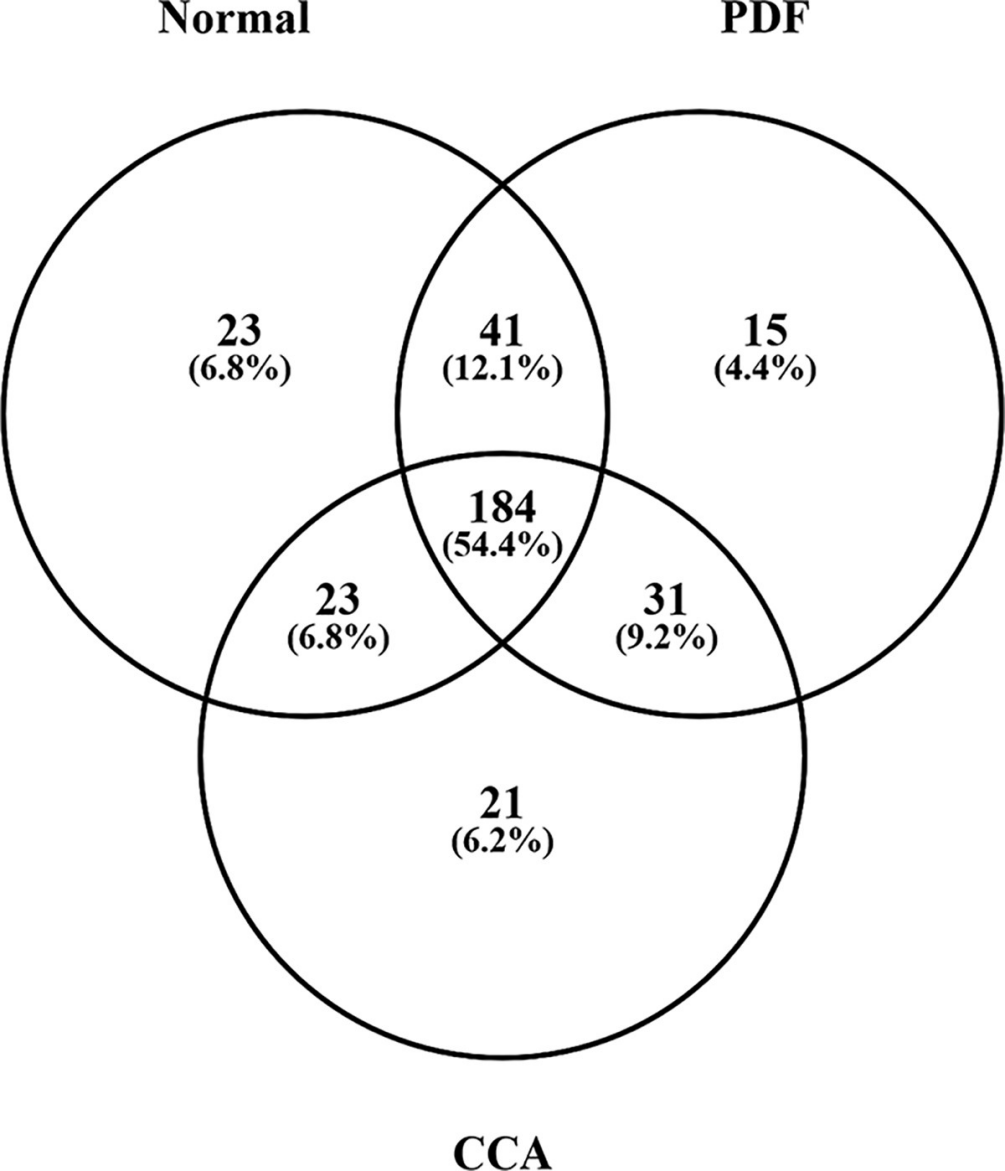
Urinary biomarker discovery. Venn diagram represents the overlap among protein sample types with 338 identified proteins.

For biomarker discovery, shotgun proteomics was conducted on pooled urine samples prepared using 300 μL each of 42 individual samples per group. Due to restrictions on mass spectrometry time, individual sample profiling could not be conducted. As the urine proteome constitute of proteins with abundance range of 10 orders of magnitudes, similar to serum protein concentration and can mask candidate biomarker detection by shotgun proteomics[28],we used an immunodepletion strategy to remove the high abundance proteins. A total of 338 proteins were identified from the three samples after immunodepletion, LC-MS/MS and database searching (S1 Table). As shown in Fig 2, 54.4% of proteins were common among the three groups (184 proteins), whereas there were 15 (4.4%) and 21 (6.2%) unique proteins in the PDF and CCA groups, respectively. From the 338 identified urinary proteins, we selected 70 candidates for qualification, with the quality requisites of one or more unique peptide detected and protein identification score of more than five. The candidates included all unique proteins in PDF and CCA groups, and those with a 1.5-fold or more change of intensity between normal and PDF, or normal and CCA (S2 Table).

### Biomarker qualification by targeted proteomic analysis

**Fig 3:**
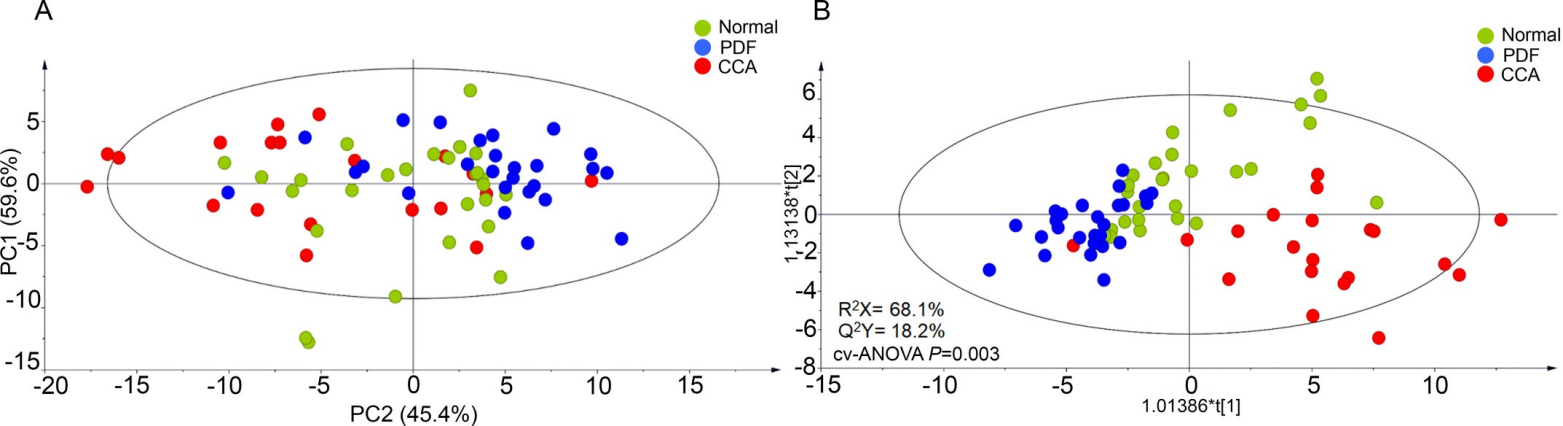
Biomarker qualification and multivariate analysis. (A) and (B) PCA and O-PLS-DA score plots of MRM results of urinary candidates that show sample differentiation; normal pathology (green), PDF (blue) and CCA group (red).

A multiple reaction monitoring (MRM) assay was developed for the 70 selected candidates as described in S1 Appendix. The transition list is available in S3 Table. This assay was used to measure the candidates in an independent cohort of 90 individual urine samples, 30 per group. Principal Components Analysis using the full data showed no obvious clustering among groups (Fig 3A). However, patient groups could be separated using supervised O-PLS-DA with Pareto (Fig 3B). The model statistics of R^2^X and Q^2^Y were 68.1% and 18.2%, respectively, indicating that the models are robust for the discrimination of statistical differences (*P* = 0.003) (Fig 3B). To identify candidate biomarkers, multivariate models were developed for pairwise comparisons, and further refined by their univariate statistics in any pairwise comparison (Table 2). While PDF could not be distinguished from normal (*P* = 0.39329), CCA could be separated from normal (*P* = 0.00075) as well as PDF (*P*<0.00001). Setting a cut-off based on Pearson correlation coefficient at p-value of 0.05 in each pairwise comparison O-PLS-DA model (df = 60, p(corr)> 0.25), as well as a cut-off of *P*<0.05 by Mann Whitney test for any univariate comparison, 27 protein candidates were selected (Table 2).

**Table 2:** The correlation coefficient values of multivariate analysis and the differences of univariate analysis of all qualified urine protein candidates using O-PLS-DA analysis.

**No.**	**Gene name**	**Protein_ID**	**Protein Name**	**O-PLS-DA Model**					
				(+)PDF vs (-)N		(+)N vs (-)CCA		(+)PDF vs (-)CCA	
				R^2^X = 53.3%		R^2^X = 49.01%		R^2^X = 60.6%	
				Q^2^Y = 7.8%		Q^2^Y = 35.2%		Q^2^Y = 47.5%	
				CV-ANOVA		**CV-ANOVA**		**CV-ANOVA**	
				*P* = 0.39329		***P = 0*.*00075***		***P<0*.*00001***	
				**p(corr)**	***P***	**p(corr)**	***P***	**p(corr)**	***P***
1	QSOX1	O00391	Sulfhydryl oxidase 1	+0.8262	**	+0.4494	***	+0.8618	****
2	CUBN	O60494	Cubilin	+0.8675	***	+0.2862		+0.7742	****
3	ALDOB	P05062	Fructose-bisphosphate aldolase B	+0.6070	*			+0.6524	**
4	LDHB	P07195	L-lactate dehydrogenase B chain	+0.5499	*	+0.2669		+0.5975	***
5	CTSD	P07339	Cathepsin D	+0.4565	*	+0.6003	**	+0.6639	****
6	HEXB	P07686	Beta-hexosaminidase subunit beta	+0.5276	**			+0.4629	*
7	CTSB	P07858	Cathepsin B	+0.4799	*				
8	NGFR	P08138	Tumor necrosis factor receptor superfamily member 16	+0.7148	*			+0.5093	**
9	CSF1	P09603	Macrophage colony-stimulating factor 1	+0.7236	**		*	+0.7864	****
10	LAMP1	P11279	Lysosome-associated membrane glycoprotein 1	+0.7553	*	-0.3105		+0.4459	
11	CDH1	P12830	Cadherin-1	+0.7765	*			+0.4845	**
12	LAMP2	P13473	Lysosome-associated membrane glycoprotein 2	+0.5142	*	-0.2945		+0.4170	
13	ANPEP	P15144	Aminopeptidase N	+0.6536	*	+0.4812	*	+0.6203	***
**No.**	**Gene name**	**Protein_ID**	**Protein Name**	**O-PLS-DA Model**					
				(+)PDF vs (-)N		(+)N vs (-)CCA		(+)PDF vs (-)CCA	
				R^2^X = 53.3%		R^2^X = 49.01%		R^2^X = 60.6%	
				Q^2^Y = 7.8%		Q^2^Y = 35.2%		Q^2^Y = 47.5%	
				CV-ANOVA		**CV-ANOVA**		**CV-ANOVA**	
				*P* = 0.39329		***P = 0*.*00075***		***P<0*.*00001***	
				**p(corr)**	***P***	**p(corr)**	***P***	**p(corr)**	***P***
14	ARSA	P15289	Arylsulfatase A	+0.6561	**	+0.3088	*	+0.6857	****
15	GNS	P15586	N-acetylglucosamine-6-sulfatase	+0.6074	***	+0.4606	**	+0.6407	****
16	CDH2	P19022	Cadherin-2	+0.3771	*	+0.3688		+0.4775	*
17	SIRPA	P78324	Tyrosine-protein phosphatase non-receptor type substrate 1	+0.7258	*			+0.7130	***
18	DPT	Q07507	Dermatopontin	+0.5355	***			+0.7207	****
19	LGALS3BP	Q08380	Galectin-3-binding protein	+0.5655	*			+0.5785	**
20	ASAH1	Q13510	Acid ceramidase	+0.6289	**			+0.6086	***
21	HAVCR2	Q8TDQ0	Hepatitis A virus cellular receptor 2	+0.7361	**			+0.6285	***
22	PVRL2	Q92692	Nectin-2	+0.5745	**	+0.5347	***	+0.7271	****
23	GGH	Q92820	Gamma-glutamyl hydrolase	+0.6948	**			+0.5754	**
**24**	**CDHR2**	**Q9BYE9**	**Cadherin-related family member 2**	+0.6854	**	**-0.4208**	*		
25	WNK1	Q9H4A3	Serine/threonine-protein kinase WNK1	+0.5186	**	+0.6782	****	+0.7182	****
26	SIAE	Q9HAT2	Sialate O-acetylesterase	+0.7384	***			+0.6408	**
27	RETN	Q9HD89	Resistin	+0.5125	*			+0.3340	

NOTE: ***P*** = *P*-values were calculated using a Mann-Whitney U -test for pairwise group comparison.

* *P*<0.05

** *P*<0.01

*** P<0.001

**** *P*<0.0001.

The p(corr) value is a correlation coefficient (ranging from -1.0 to 1.0) for each model.

“+” or “–”indicates higher correlation in either group of O-PLS-DA pairwise comparison models.

The *P*-value of all O-PLS-DA models was derived from permutation tests (n = 500).

### Protein interaction network of urinary candidate proteins

**Fig 4:**
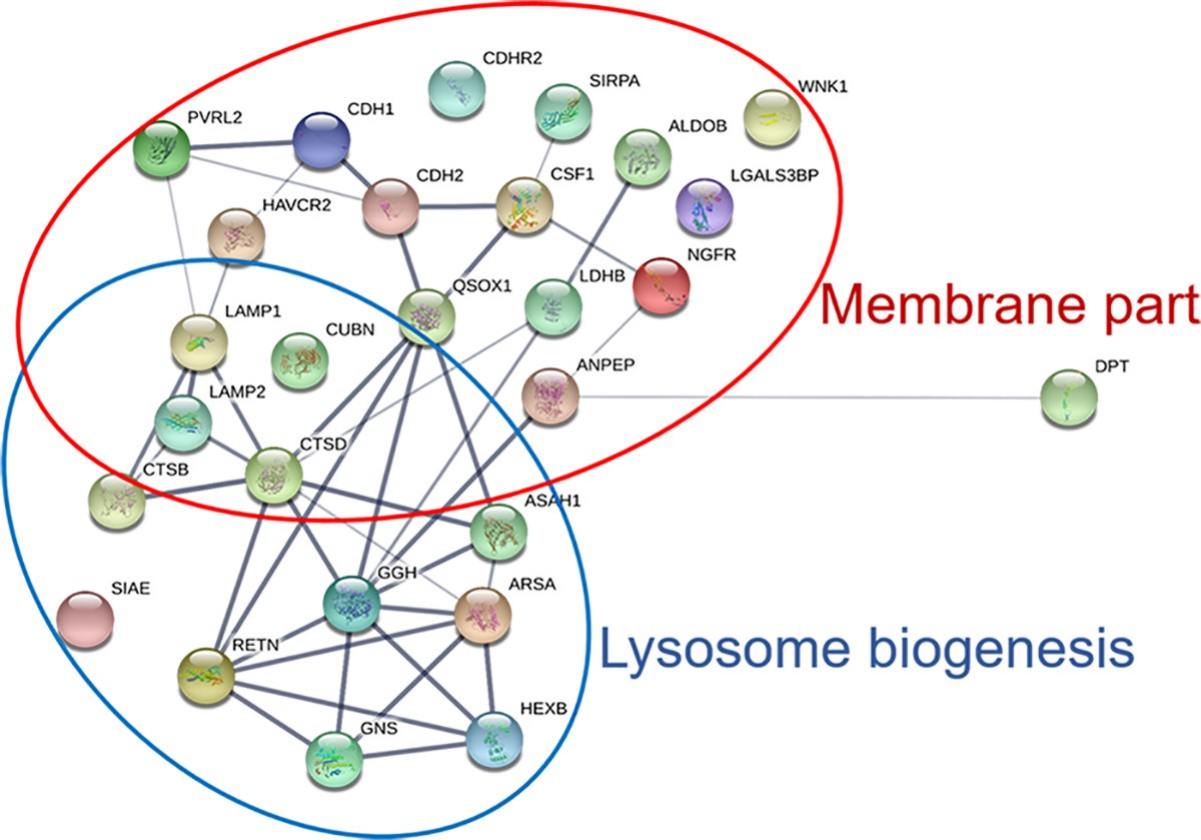
The protein interaction network analysis of 27 significant candidate proteins using the STRING database (http://string-db.org). A; two main cellular component pathways were identified in this study: lysosome biogenesis (blue circle) and membrane part (red circle). Each node lists the gene name of the candidate according to protein ID from Table 2. The different intensity of lines represents the protein association of confidence.

To evaluate any functional and/or physical interactions between the urinary protein candidates, a protein interaction network was constructed for the 27 candidates using STRING and functional gene ontology (GO) enrichment analysis (Fig 4, S4 Table). This analysis revealed that the majority of urinary candidate proteins were membrane proteins and/or associated with the biological processes of the lysosome biogenesis.

### Orthogonal validation of potential biomarkers using immunohistochemical staining

**Fig 5:**
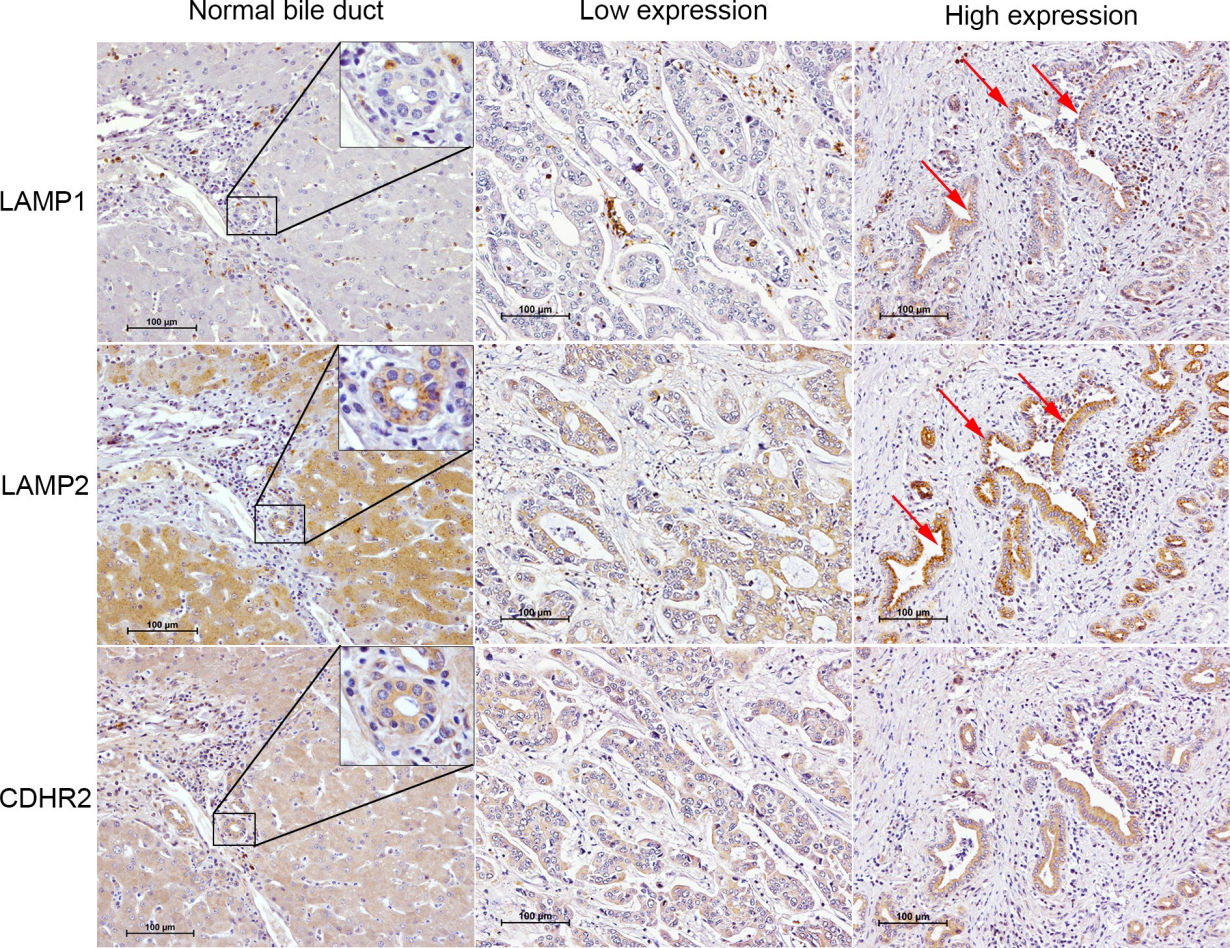
Immunohistochemical staining of three candidate proteins. LAMP1, LAMP2 and CDHR2, was performed on cadaveric donor liver tissues (the first column) and human CCA microtissue arrays which demonstrated low and high expression (the second and third column). The red arrows indicate the positive of LAMP1 and LAMP2 expression at the luminal surface (red arrows).

As an independent validation for potential CCA biomarkers, we chose 3 candidates that were elevated in CCA in the normal versus CCA comparison (negative p(corr) value in Table 2), lysosome associated membrane glycoproteins 1 (LAMP1), lysosome associated membrane glycoproteins 2 (LAMP2) and cadherin-related protein member 2 (CDHR2). For immunohistochemistry validation, we developed a human CCA tissue microarray containing more than 200 specimens, and compared with a limited number of cadaveric liver tissues. All three antibodies were strongly positive in human CCA tissues when compared with the normal bile duct in cadaveric liver donor tissues (Fig 5), with more than 50% of cores showing high expression level for each of the 3 candidate proteins (Table 3). Moreover, high expression of LAMP2 significantly correlated with late stage CCA based on TNM staging (*P*<0.05) (Table 3). LAMP1 and LAMP2 were expressed in the cytoplasm of cancer cells and inflammatory cells, whereas CDHR2 was only expressed in the cytoplasm of cancer cells (Fig 5). Interestingly, high expression of LAMP1 and LAMP2 was also detected at the luminal surface, potentially indicative of lysosome formation (Fig 5). These results provide additional evidence for urinary LAMP1, LAMP2 and CDHR2 as potential CCA biomarkers.

**Table 3:** Clinico-pathological data and urinary candidate expression.

	LAMP1 expression			LAMP2 expression			CDHR2 expression		
	L	H	*P*-value	L	H	*P*-value	L	H	*P*-value
**Sample size**	89	121		85	124		99	114	
**Gender**			0.656			0.183	67 32	75 39	0.884
Male	58	83		52	87		67	75	
Female	31	38		33	37		32	39	
**Age (year)**			0.405			0.674			0.585
<61	45	54		41	56		44	55	
≥61	44	67		44	68		55	59	
^**‡**^**BUN (cut off >19.1 mg/dL)**			0.125			0.054			0.714
Normal	66	80		63	83		72	77	
Abnormal	4	1		0	5		2	3	
^**‡**^**Creatinine (cut off >1.5 mg/dL)**			0.882			0.492			0.937
Normal	68	79		62	85		72	78	
Abnormal	4	2		1	3		2	2	
^**‡**^**CA19-9 (cut off >100 μg/mL)**			0.868			0.868			1.000
Normal	27	33		26	35		29	32	
Abnormal	43	48		37	53		45	48	
^**‡**^**CEA (cut off >5 ng/mL)**			0.510			0.101			0.333
Normal	29	47		27	50		34	44	
Abnormal	41	34		36	38		40	36	
**Tumor type**			1.000			0.485			0.217
Papillary	44	59		44	58		44	61	
Non-papillary	45	62		41	66		55	53	
**Histological type**			0.161			0.066			0.782
Extraductal CCA	35	60		31	62		45	49	
Intraductal CCA	54	61		54	62		54	65	
**TNM stage**			0.569			**0.021**			0.575
Early stage	38	46		42	41		42	43	
Late stage	51	75		43	83		57	71	
**Metastasis status**			0.886			0.147			0.673
Negative	33	47		38	42		36	45	
Positive	56	74		47	82		63	69	
**Survival status**			0.750			0.747			0.747
Alive	21	31		20	35		20	32	
Death	68	90		65	92		65	92	

NOTE: *P*-value were calculated using Pearson’s chi-square test.

^‡^indicating there are missing data.

L, Low and H, High expression.

## Discussion

This study reports the discovery and qualification of potential CCA urinary biomarkers for CCA screening in Ov-associated PDF. The workflow comprised a discovery phase using shotgun proteomics (LC-MS/MS), a qualification phase with targeted proteomics (MRM-MS) and a validation phase with tissue immunohistochemistry. In the Ov-associated CCA endemic area, periductal fibrosis (PDF) is recognized as a precancerous lesion and sonographic marker for CCA development. This study is the first to use urine sample of sonography-diagnosed PDF patients as an additional comparison group for early CCA detection.

Out of the 338 urinary proteins discovered, 70 (21%) were selected for the qualification phase using relatively non-stringent criteria. Qualification data obtained by MRM-MS analysed by multivariate analysis (O-PLS-DA) indicated that a subset of the urine proteome could discriminate patients with CCA compared to normal and PDF subjects. In addition, pairwise PCA and O-PLS-DA comparison analyses were also performed and the results demonstrated that the urinary candidates showed a slight grouping when the normal and PDF groups were compared in either model (S1 Fig). Combining multivariate and univariate statistics which multivariate analysis were developed for selecting protein candidates and further refined using univariate statistics in an individual candidate in any pairwise comparison, we were able to qualify 27 urinary candidates for detection of CCA from either normal or PDF subjects.

Based on the MRM analysis, three urinary protein candidates showing higher correlation with CCA were chosen for tissue-based validation. By IHC, we confirmed the protein expression levels of LAMP1, LAMP2 and CDHR2 are high in human CCA tissues when compared with cadaveric liver donor tissues. Although the number of liver donor tissue were limited, detection of these candidates in CCA tissues provide additional support for these candidate biomarkers.

LAMP1 and LAMP2 are major protein components of the lysosomal membrane and are involved in lysosome biogenesis and degradation in order to maintain metabolic homeostasis [29]. In addition to their role in lysosomal processing, LAMP1 and LAMP2 are associated with autophagy biogenesis [30]. However, autophagy can promote cancer cells to survive under stress conditions and during chemotherapeutic treatment via enhancing autophagy [31]. Many reports show that LAMP1 and LAMP2 are implicated in promoting cancer progression [32, 33]. High LAMP1 expression has been found in cancer development, progression tumor metastasis in astrocytoma, colorectal cancer, pancreatic carcinoma and various other cancer tissues [34–36]. Our tissue IHC staining data demonstrate that LAMP1 and LAMP2 expression is highly positive at the apical site of tumor cells (Fig 5). Based on the STRING network analysis, these candidate proteins are known to be associated with extracellular exosome, extracellular region, cytoplasmic membrane-bound vesicle and endocytic vesicle which also involved in lysosome biogenesis. As both LAMP1 and LAMP2 proteins have been reported to associate with increasing autophagic vacuole accumulation and altered lysosomal formation[30], our results suggest a link of autophagy in CCA development via LAMP1 and LAMP2 expression. In support of this, Thongchot and co-workers reported that hypoxia associated autophagy promotes CCA progression, leading to high mortality rates in CCA patients [37].

Cadherin-related family member 2, also known as protocadherin-24 (PCDH24) or proto-cadherin liver kidney and colon protein (PCLKC), plays an important role in contact inhibition at the lateral surface of epithelial cells [38]. Okazaki and co-workers reported for colorectal cancer cell that PCDH24 can act as a tumor suppressor by inhibiting tumor formation that induces contact inhibition [38]. In contrast, we found higher CDHR2 in CCA patient urine and highly positive staining for CDHR2 in human CCA tissues. This is the first report on the potential involvement of CDHR2 in CCA.

## Conclusion

In conclusion, we report the discovery and validation of candidate urinary biomarkers for CCA compared with the normal and PDF groups. LAMP1, LAMP2 and CDHR2 are the chosen potential biomarkers for CCA detection that were confirmed using IHC techniques. The three potential biomarkers discovered in this study using urine samples, could provide a suitable system for the early diagnosis of CCA either in combination with or as a replacement for serum/plasma or bile fluid analysis. This non-invasive technique is likely to be more useful for the screening and monitoring for early CCA detection and surveillance. However, these candidates still need to be further evaluated using a larger set of independent patient cohorts.

## Supporting information

S1 Appendix Data processing and normalization for MRM analysis. (DOCX)

S1 Table All of total identified proteins in discovery phase using MaxQuant software searching. (XLSX)

S2 Table The urinary candidates list from the discovery phase following criteria selection for MRM-MS experiment. (XLSX)

S3 Table All of peptides and transition of selected serum candidate proteins for the MRM-MS experiment. (XLSX)

S4 Table The functional gene ontology (GO) enrichment analysis of the verified urinary candidates using STRING software (http://string-db.org) to predict protein-protein associations with other proteins in different pathways. (XLSX)

S1 Fig Multivariate analysis with pairwise comparison of individuals urine samples in qualification phase including PCA and O-PLS-DA analysis. (TIF)
