# Supplementary material for: Urine proteomics study reveals potential biomarkers for the differential diagnosis of cholangiocarcinoma and periductal fibrosis
Source: PLoS One. 2019 Aug 19;14(8):e0221024. doi: 10.1371/journal.pone.0221024 (PMC6699711; doi:10.1371/journal.pone.0221024)
Supplement: S1 Appendix — (DOCX) [file pone.0221024.s001.docx]

**S1 Appendix. Data processing and normalization for MRM analysis**

**Data processing for MRM experiment development**

During MRM method development, urine samples from each group were combined and injected. Multiple MRM methods consisting of a maximum of 200 transitions were created as a first step of method development. These were transferred across to Skyline software version 3.7.1.11208 on August 2017 (http://skyline.maccosslab.org/) for visualization, subsequent method refinement and analysis [1]. Using MS/MS discovery data, a spectral reference library was constructed in Skyline. Pooled urine samples from each of the group of samples, including normal, PDF ultrasound and CCA groups, were combined and run for each un-scheduled method to identify the best MRM transitions to obtain the protein candidate list. Each method incorporated transitions for internal standard chicken ovalbumin and indexed retention time (iRT) peptides. Retention time prediction calculator iRT-C18 of Skyline was used to increase confidence in peptide identification [2]. The iRT scale was used to calibrate the retention time of the known peptides which is based on the calibration plot so that the retention time of the peptide of interest can be predicted. All urine candidate proteins passed the protein selection criteria for MRM analysis of 2–3 peptides per protein and 2–3 transitions per peptide. Raw data from the MRM-MS experiment were processed in Skyline. Using retention time information for each peptide, one final scheduled MRM method was created incorporating a total of 66 proteins (2 proteins were included for the internal control including iRT peptides and ovalbumin), 187 peptides and 561 transitions with a retention times window of 2 min (S3 Table). All peaks were manually checked for correct integration, and peak area for each peptide (sum of all transitions) was exported for further analysis.

**Data normalization for MRM analysis**

Area under the curve of each peptide was exported from Skyline and represented as the peak intensity of the peptide which was normalized using the median of normalized area under peak of iRT peptide. The normalized peptides having correlations of >0.8 using Pearson’s correlation test were selected for further analysis. Then, normalized peptide intensity was determined to convert from peptide to protein level and a log2 transformation was performed to obtain a near-normal distribution needed for statistical tests using in R software. The N/A value were replaced as minimum values the lowest of normalized intensity peptides.

**References**

1. MacLean B, Tomazela DM, Shulman N, Chambers M, Finney GL, Frewen B, et al. Skyline: an open source document editor for creating and analyzing targeted proteomics experiments. Bioinformatics. 2010;26(7):966-8. doi: 10.1093/bioinformatics/btq054. PubMed PMID: 20147306; PubMed Central PMCID: PMCPMC2844992.

2. Escher C, Reiter L, MacLean B, Ossola R, Herzog F, Chilton J, et al. Using iRT, a normalized retention time for more targeted measurement of peptides. Proteomics. 2012;12(8):1111-21. doi: 10.1002/pmic.201100463. PubMed PMID: 22577012; PubMed Central PMCID: PMCPMC3918884.
