# Supplementary figures and images for: Urine proteomics study reveals potential biomarkers for the differential diagnosis of cholangiocarcinoma and periductal fibrosis
Source: PLoS One. 2019 Aug 19;14(8):e0221024. doi: 10.1371/journal.pone.0221024 (PMC6699711; doi:10.1371/journal.pone.0221024)

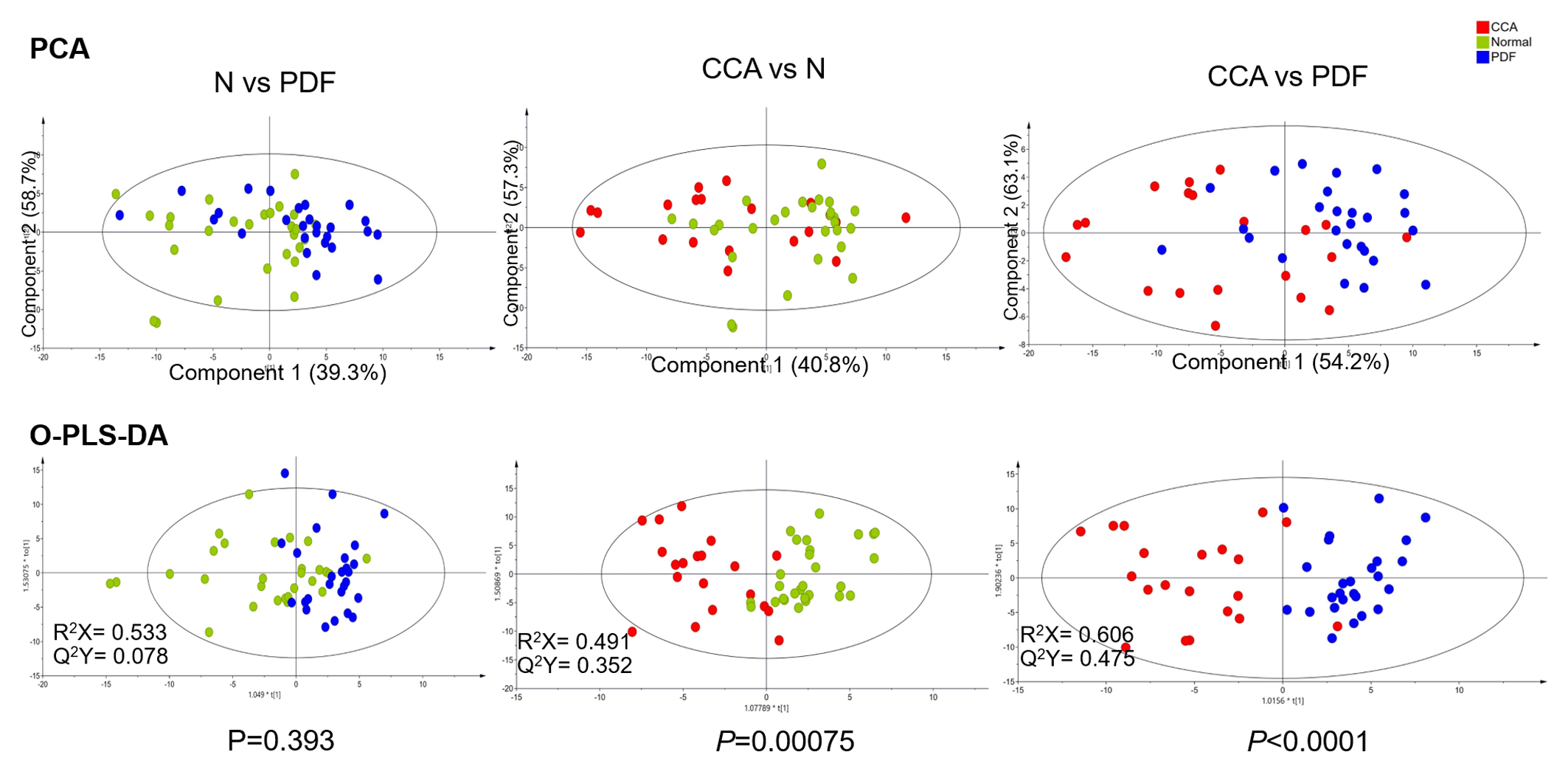

Supplement: S1 Fig — (TIF) [file pone.0221024.s006.tif]
